# Incidence rates of suicide attempts and self-harm in Europe. What can we learn? A systematic review and meta-analysis

**DOI:** 10.1192/j.eurpsy.2022.2179

**Published:** 2022-09-01

**Authors:** S. Jakobsen, E. Christiansen, P. Andersen, J. Lauritsen, E. Stenager

**Affiliations:** 1 University of Southern Denmark, Department Of Regional Health Research, Odense M, Denmark; 2 University of Southern Denmark, Department Of Public Health, Esbjerg, Denmark; 3 Odense University Hospital, Department Of Orthopedics And Traumatology, Odense, Denmark; 4 The Region of Southern Denmark, The Department Of Psychiatry, Aabenraa, Denmark

**Keywords:** suicide attempt, self-harm, Prevalence, Definition

## Abstract

**Introduction:**

Definitions used for suicide attempts and self-harm have been discussed for many years and is used differently in European countries, sometimes even interchangeably. Therefore, it is difficult to compare relevant rates across nations.

**Objectives:**

This study aims at estimating the rate of suicide attempts and self-harm in chosen European countries in the more recent years when distinguishing between applied definitions.

**Methods:**

A systematic search for relevant articles published between 2010-2020 will be performed in databases such as PubMed, Embase, PsycINFO, and Web of Science. Only articles in English or Danish will be included. Data will be collected for all age groups above 15 years of age. The prevalence of suicide attempts and self-harm will be calculated by a random effect model. Subgroup analyses will be performed to compare the rates according to age.

**Results:**

from the performed systematic review and meta-study will be presented at the conference.

**Conclusions:**

The conclusion will be presented when results have been analysed.

**Disclosure:**

No significant relationships.

